# Adipogenic Activity of Wild Populations of *Rhododendron groenlandicum*, a Medicinal Shrub from the James Bay Cree Traditional Pharmacopeia

**DOI:** 10.1155/2015/492458

**Published:** 2015-10-05

**Authors:** Michel Rapinski, Lina Musallam, John Thor Arnason, Pierre Haddad, Alain Cuerrier

**Affiliations:** ^1^Canadian Institutes of Health Research Team in Aboriginal Antidiabetic Medicines, Université de Montréal, Montréal, QC, Canada H3C 3J7; ^2^Institut de Recherche en Biologie Végétale, Jardin Botanique de Montréal, Université de Montréal, 4101 Sherbrooke Est, Montréal, QC, Canada H1X 2B2; ^3^Natural Health Products and Metabolic Diseases Laboratory, Department of Pharmacology, Université de Montréal, Montréal, QC, Canada H3C 3J7; ^4^Centre for Research in Biotechnology and Biopharmaceuticals, Department of Biology, University of Ottawa, Ottawa, ON, Canada K1N 6N5

## Abstract

The traditional medicinal plant, Labrador tea (*Rhododendron groenlandicum* (Oeder) Kron & Judd; Ericaceae), present in the pharmacopoeia of the Cree of Eeyou Istchee, has shown glitazone-like activity in the 3T3-L1 adipogenesis bioassay. This activity has been attributed to phenolic compounds, which have been shown to vary in this plant as a function of insolation parameters. The goal of this study was to determine if these changes in phenolic content were pharmacologically significant. Leaves were harvested in 2006 throughout the James Bay region of Northern Quebec and ethanol extracts were tested *in vitro* using the 3T3-L1 murine cell line adipogenesis bioassay. This traditional medicinal plant was found active in the assay. However, there was no detectable spatial pattern in the accumulation of intracellular triglycerides, suggesting that such patterns previously observed in the phenolic profile of Labrador tea were not pharmacologically significant. Nonetheless, a reduction in the adipogenic activity was observed and associated with higher concentrations of quercetin for which selected environmental variables did not appropriately explain its variation.

## 1. Introduction

In a previous study on the phytochemistry of the North American medicinal plant,* Rhododendron groenlandicum* (Oeder) Kron & Judd (Ericaceae), Labrador tea, we found the concentration of biologically active compounds to vary in Northern Quebec's Hudson and James Bay region [1]. Labrador tea is a common species in Canada's boreal forest. More importantly, it is a popular medicinal plant found in the traditional pharmacopoeia of indigenous populations from the Algonquian, Salish, Wakashan, Tsimshian, and Eskimo-Aleut linguistic families [2–9].

In ethnobotanical studies conducted in six communities of the Cree Nation of Eeyou Istchee (CEI), we found* R. groenlandicum* to be the top-ranked plant species used for the treatment of symptoms associated with type 2 diabetes (T2D) [10–13]. The inherent cultural relevance of this species to CEI traditional medicine (CTM) warrants further investigation into its antidiabetic potential.

The CIHR Team on Antidiabetic Aboriginal Medicines (CIHR-TAAM), formed through collaborative work between CEI communities, the Cree Board of Health and Social Services of James Bay (CBHSSJB), and Canadian academic researchers, has screened many of the multiple plants present in the CEI pharmacopoeia [14–17]. Of these,* R. groenlandicum* was shown to possess* in vitro* glitazone-like activity comparable to rosiglitazone in an adipogenic assay measuring the lipid accumulation in differentiating 3T3-L1 preadipocytes [14].

The antidiabetic drug rosiglitazone induces an increase in the sensitivity to insulin, acting as a PPAR*γ* receptor agonist [18]; the expression of this transcription factor is particularly implicated in the differentiation of adipocytes [19–22] and in insulin sensitivity [18]. Hence, it plays a critical role in the pathogenesis of T2D. The action of PPAR*γ* results in an improvement in the absorption of fatty acids in differentiated adipocytes which store them as triglycerides (TG) [18]. Adipocytes therefore provide storage for fatty substances that would otherwise accumulate in tissues such as skeletal muscle and liver, thereby contributing to metabolic disorders such as insulin resistance [22].

The pharmacological activity of* R. groenlandicum* has been attributed to phenolic compounds [14, 16, 23]. Bioassay-guided fractionation using adipogenesis of 3T3-L1 murine cells confirmed that specific phenolics are the most active compounds [24]. In developing culturally appropriate approaches to treating T2D in the CEI communities, the variation of these compounds in* R. groenlandicum* has important implications in ensuring the quality control of traditional medicinal plants or to develop standardized natural health products (NHPs).

In this study, we assessed possible variations in the antidiabetic potential of* R. groenlandicum*. Our objective was to determine if the phytochemical variations observed in the species' phenolic profile are biologically significant. We evaluated the* in vitro* pharmacological activity of crude extracts from various localities using the adipogenesis bioassay and hypothesized that high concentrations of phenolic compounds would result in a stronger adipogenic activity.

## 2. Materials and Methods

### 2.1. Sampling, Extraction, and Phytochemical Analysis

The sampling, extraction, and analytical methods for phytochemical identification and quantification are thoroughly described in Rapinski et al. [1], which reports on the phytochemistry of* R. groenlandicum.* A subsample, selected randomly, of previously reported samples was used in this* in vitro* study.

Briefly, mature leaves were sampled during the summer of 2006 around the communities of Mistissini, Nemaska, Eastmain, Wemindji, and Whapmagoostui, thus covering much of the northsouth gradient in Eeyou Istchee. Five accessions, each containing leaves from multiple individual plants, were collected within a 50 km radius around each community and selected for this study. Samples were air-dried and preserved in paper bags at room temperature.

Samples were milled through a Wiley Mill at 40 mesh and extracted overnight in 25 mL/g of 80% EtOH by orbital shaking at room temperature at 250 RPM. The pellet was extracted overnight in 15 mL 80% EtOH. An aliquot (1 mL) of the pooled supernatants (adjusted to 50.0 mL in a volumetric flask) was prepared for High Performance Liquid Chromatography coupled with Diode Array Detector (HPLC-DAD). The leftover crude extracts were dried using a speedVac; the trace water was removed by lyophilization using SuperModulo freeze dryer and the dehydrated extracts were stored at −80°C.

Finally, 10 *μ*L of each extract aliquot was injected through an autosampler and detected by DAD at 290 nm, bandwidth 4, reference off. The separations were performed on a Luna C18 column (250 × 4.6 mm, 5 *μ*M particle size). Peak identification was undertaken by cochromatographic comparison of the spectral data adopted in our in-house metabolomics spectral library [23]. A standard curve was constructed by injection of serially diluted marker compounds in methanol. The quantification was based on peak area. The quantitation of putatively identified quercetin-glycosides was achieved based on calibration curve of quercetin-3-galactoside. Each sample was analyzed in triplicate and averaged to account for instrumental variation.

### 2.2. Cell Culture

3T3-L1 murine preadipocyte cells were grown to confluence in 24-well plates in DMEM proliferation medium containing 10% FBS. Media were changed every 2 days. At 24 h after confluence (day 0), cells were induced to differentiate with a short-term differentiation medium of DMEM supplemented with 10% FBS, 1 *μ*M DMX, 250 *μ*M IBMX, and 500 nM insulin. After 48 h, the media were replaced with DMEM containing 10% FBS and 500 nM insulin for long-term differentiation. Cells were differentiated for a total of 5 days with media change every 2 days.* Rhododendron groenlandicum* crude extracts (75 *μ*g/mL) and rosiglitazone (10 *μ*M; positive control) were dissolved in DMSO and added to the cells as of day 0 of differentiation. The final concentration of DMSO was kept at 0.1% throughout the differentiation period.

### 2.3. Adipogenesis

We measured intracellular TG content at day 5 of differentiation using the AdipoRed reagent according to the manufacturer's instructions. Methods have been previously described in Spoor et al. [14] and Harbilas et al. [16]. In short, wells containing adipocytes were washed twice with phosphate-buffered saline (PBS) before 1 mL of PBS containing 30 *μ*L of AdipoRed reagent was added to each well and incubated for 15 minutes at room temperature. AdipoRed becomes fluorescent when partitioned in a hydrophobic compartment, namely, intracellular triglycerides (TG). The fluorescence of each well was measured with a Wallac Victor2 fluorimeter (Perkin-Elmer, Saint-Laurent, QC) at an excitation wavelength of 485 nm and an emission wavelength of 572 nm. The results were reported as percentage of the vehicle control, 0.1% DMSO.

### 2.4. Cell Lines, Chemicals, Biochemicals, and Standards

For the identification and quantification of phenolic markers, (+)-catechin (**1**), chlorogenic acid (**2**), (−)-epicatechin (**3**),* p-*coumaric acid (**4**), rutin (**5**), quercetin-3-galactoside (**6**), quercetin-3-glucoside (**7**), quercetin-3-rhamnoside (**12**), myricetin (**13**), and quercetin (**14**) were purchased from Sigma-Aldrich (Oakville, Ontario, Canada) and Extrasynthese (Genay, France). HPLC grade water, acetonitrile, and formic acid (99% purity) were purchased from Sigma-Aldrich.

For cell culture and adipogenesis, preadipocyte 3T3-L1 cell line was purchased from the American Type Culture Collection (ATCC; Manassas, VA). Dexamethasone (DMX), bovine pancreatic insulin, 3-isobutyl-1-methylxanthine (IBMX), and Dimethyl sulfoxide (DMSO) were purchased from Sigma-Aldrich (Oakville, ON). Rosiglitazone was obtained from Alexis Biochemicals (Hornby, ON).

Dulbecco's Modified Eagle Medium (DMEM), fetal bovine serum (FBS), and bovine calf serum (NCS) were from Wisent Inc. (Saint-Bruno, QC). AdipoRed reagent was purchased from Cambrex Bio Science Walkersville Inc. (Walkersville, MD).

### 2.5. Environmental Data

Annual estimates of bioclimatic variables, namely, annual temperature range, corresponding to our sampling year and long-term estimates for insolation variables were provided by the Canadian Forest Services of Natural Resources Canada [25]. Long-term estimates were derived from multidecade meteorological data collected from 1971 to 2000 [26, 27].

### 2.6. Statistical Analysis

To reduce interassay variation, TG content was normalized relative to each assay's vehicle control, 0.1% DMSO, set at 100%.* Rhododendron groenlandicum* and rosiglitazone always induced significant increases in activity as verified by the fact that the 95% confidence interval of the mean activity (quadruplicate determinations) did not include the 100% adipogenic activity reference (*p* < 0.05). Differences between communities were analyzed by one-way analysis of variance. The relationships between TG content and compounds were analyzed by multiple and simple linear regressions. To represent the adipogenic activity of* R. groenlandicum* and the quantified compounds, principal components analysis (PCA) was performed on the matrix of these compounds using the correlation matrix. Individual samples were scored onto the PCA axes and represented with the vectors for each compounds. TG content was subsequently projected as a supplementary variable onto the principal components in order to interpret the dimensions of variability. In doing so, the calculation of distances between each of the samples and the construction principal components depends only on their phytochemical profile. Using the transition formulae described by Lê et al. [28], the coordinates for TG content are calculated using the original eigenvalues. Finally, we partitioned the variation in the adipogenic activity of* R. groenlandicum* between the two sets of variables: compounds and environmental factors. This was done using a partial-redundancy analysis (partial-RDA) approach [29, 30]. All analyses were performed using R statistical language [31]. Results are reported as means ± SD and statistical significance is set at *α* = 0.05.

## 3. Results and Discussion

The phytochemistry of the same* R. groenlandicum* accessions has already been described and discussed in greater length in Rapinski et al. [1]. Here, we present the results of a subsample of 2006 accessions in the adipogenesis bioassay.

**Figure 1:**
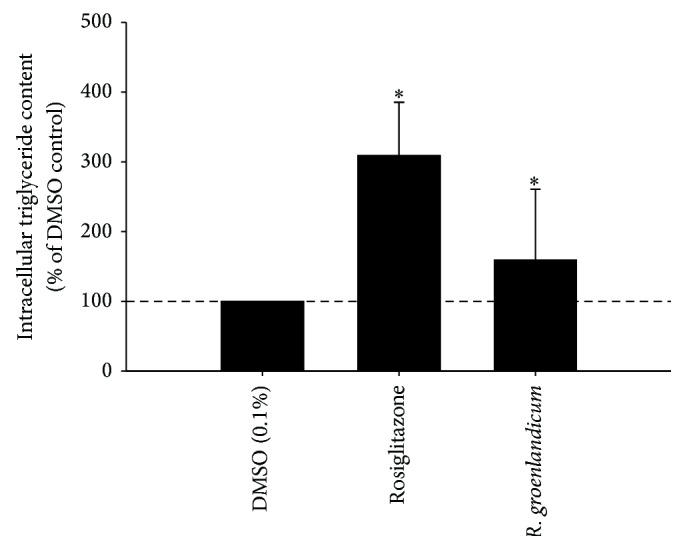
Effect of* R. groenlandicum* crude leaf extracts from Northern Quebec on lipid accumulation. Intracellular triglyceride content was measured by AdipoRed fluorescence, in live 3T3-L1 murine adipocytes incubated with plant extracts for 5 days after differentiation. Means ± SD (*n* = 4 for rosiglitazone, *n* = 100 for* R. groenlandicum*) are normalized to the vehicle control (0.1% DMSO). Asterisk (*∗*) indicates significant differences with respect to the DMSO control at *α* = 0.05.

**Figure 2:**
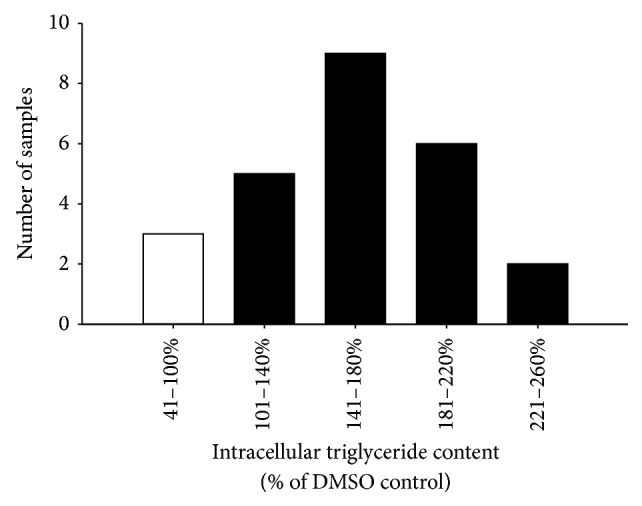
Frequency distribution of the adipogenic activity from 25 samples of* R. groenlandicum* leaves collected throughout Northern Quebec. Intracellular triglyceride content was measured by AdipoRed fluorescence, in live 3T3-L1 murine adipocytes incubated with plant extracts for 5 days after differentiation. Triglyceride content was normalized to the vehicle control (0.1% DMSO). Samples with content levels below 100% (in white) were considered inhibitory and decreased lipid accumulation.

The glitazone-like activity of* R. groenlandicum* to increase the accumulation of intracellular TG in 3T3-L1 adipocytes was measured at day 5 of differentiation. Extracts increased adipogenesis, with an average content of TG of 159.0% that of DMSO (Figure 1) and a 95% confidence interval of 138.8–179.1% of DMSO. The adipogenic activity of* R. groenlandicum* was roughly half of the positive control, rosiglitazone. This is lower than what has previously been reported for this species. Spoor et al. [14] reported the stimulation of adipogenesis to be comparable to rosiglitazone, while later determinations measured an activity representing two-thirds that of the antidiabetic drug [24]. With few exceptions (Figure 2), our results nonetheless confirm the adipogenic potential of this species. It is important to consider the fact that previous determinations of activity were carried out using extracts prepared from large quantities of source material (large number of individual plants) collected a few years prior to the material used in the present studies. Hence, interindividual variations were absent and different climatic conditions may have prevailed. This can explain, at least in part, the differences in adipogenic potential observed between the studies.

**Figure 3:**
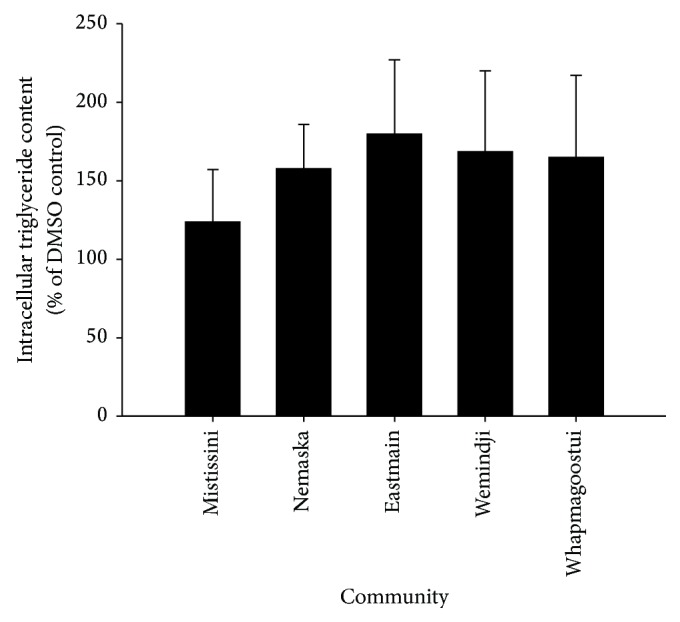
Effect of* R. groenlandicum* crude leaf extracts prepared from accessions collected around five communities in Northern Quebec on lipid accumulation. Intracellular triglyceride content was measured by AdipoRed fluorescence, in live 3T3-L1 murine adipocytes incubated with plant extracts for 5 days after differentiation. Means ± SD (*n* = 5) are normalized to the vehicle control (0.1% DMSO). There were no significant differences between communities (*p* = 0.348).

There were no statistically distinct spatial patterns detected in the pharmacological activity of* R. groenlandicum*. None of the communities sampled possessed accessions which significantly increased intracellular TG more than the others (*p* = 0.348, Figure 3). We have previously found that biologically active phenolics were greater in collections made around the communities of Nemaska, Eastmain, and Wemindji [1]. The adipogenic activity of* R. groenlandicum* followed a similar trend, as can be observed in Figure 3, although statistical significance of a polynomial relationship was not achieved (*p* = 0.170), possibly due to high variability. This suggests that variations in the phytochemical profiles, observed in Rapinski et al. [1], may be pharmacologically relevant, but further studies will be necessary to confirm this point.

**Figure 4:**
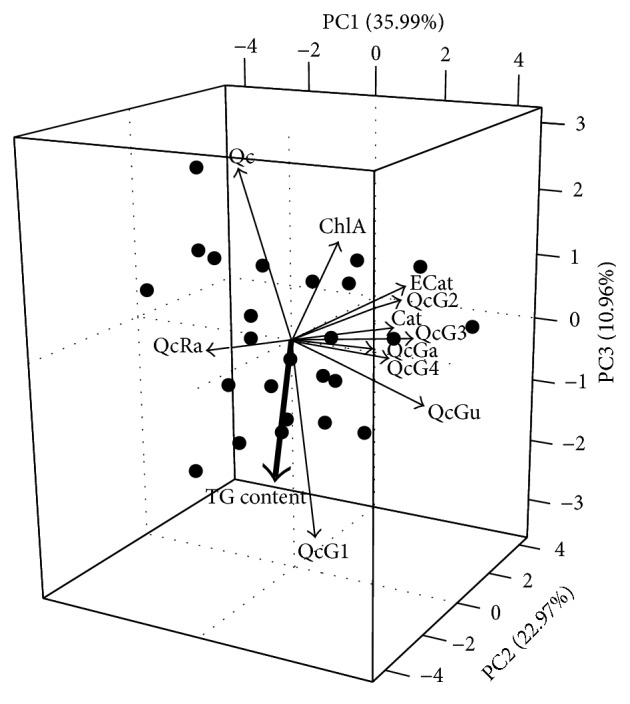
Principal component analysis biplot of 11 phenolic compounds in* R. groenlandicum* leaves. Solid lines represent relative loadings of these variables on axes 1, 2, and 3. TG content (bold arrow) was selected as a supplementary variable and plotted onto principal components generated from the phytochemical markers. Scores for individual samples are represented by symbols for the communities of Mistissini (▲), Nemaska (●), Eastmain (■), Wemindji (◆), and Whapmagoostui (▼). Abbreviations represent compounds as follows: (+)-catechin: Cat; chlorogenic acid: ChlA; (−)-epicatechin: ECat; quercetin-3-galactoside: QcGa; quercetin-3-glucoside: QcGu; quercetin-glycoside 1: QcG1; quercetin-glycoside 2: QcG2; quercetin-glycoside 3: QcG3; quercetin-glycoside 4: QcG4; quercetin-3-rhamnoside: Qc-Ra; and quercetin: Qc.

**Figure 5:**
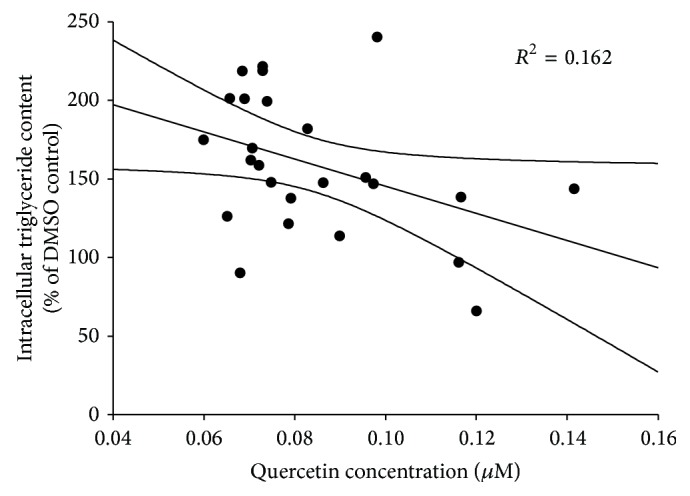
Intracellular triglycerides content of 3T3-L1 murine adipocytes exposed to 75 *μ*g/mL of* R. groenlandicum* leaf extracts collected from various locations. Quercetin concentrations in crude extract were significantly and negatively associated with the species' adipogenic activity (*p* = 0.0458). Triglyceride contents are normalized to the vehicle control (0.1% DMSO).

Indeed, we found that quantified compounds explained considerable variability obtained in this species' pharmacological activity (*p* = 0.0279, *R*_adj_^2^ = 0.491). The distribution of* R. groenlandicum* samples based on their phytochemical profile was reconstructed into a reduced three-dimensional space, which represented 69.92% of the samples' variation over three statistically constructed principal components, or axes (Figure 4). Each principal component, from the first to the third, respectively, explained 35.99% (*λ*_1_ = 3.96), 22.97% (*λ*_2_ = 2.53), and 10.96% (*λ*_3_ = 1.20) of the variation. The direction and proximity of arrows for some major markers suggest that these are highly correlated (Figure 4). When projecting intracellular TG onto this plot, it did not appear to be well correlated with the bulk of these markers, many of which were found near a 90° angle from it, thus indicating week or null correlations. One of the only markers for which a significant relationship with TG appears to exist is quercetin (Figures 4(b) and 4(c)). This suggests that, out of all the compounds assessed, variations in the adipogenic activity of* R. groenlandicum* are most vulnerable to changes in the content of quercetin found in the crude extracts.  Figure 5 further illustrates the linear correlation (*p* = 0.0458, *R* ^2^ = 0.162) whereby the adipogenic activity of* R. groenlandicum* decreases as the concentration of quercetin in the sample increases. This is consistent with observations from our own group [24] where pure quercetin was found to inhibit adipogenesis in a dose-dependent manner. The activity of quercetin is well studied and has also been consistently shown by others to be a potent inhibitor of adipocyte differentiation and adipogenesis [18, 32–34].

**Figure 6:**
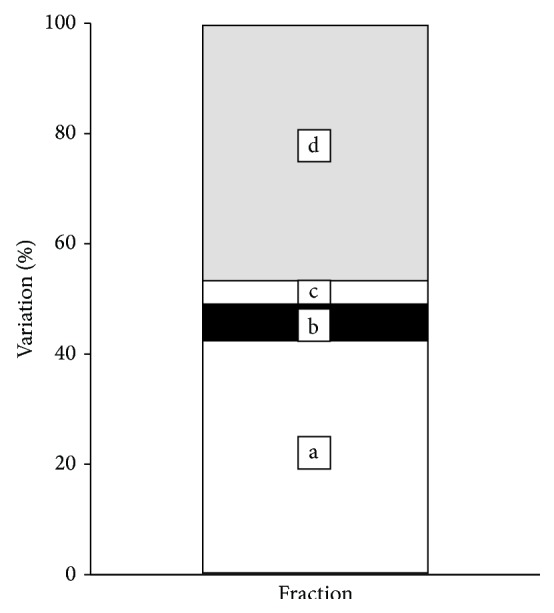
Variation partitioning of the adipogenic activity of* R. groenlandicum* leaf extracts explained by the content in biologically active compounds and the effect of bioclimatic variables. *a* = compounds∣environment, *b* = shared component, *c* = environment∣compounds, and *d* = unexplained (see Table 1 for more details). The full model, fraction [*a* + *b* + *c*], as well as the model including compounds only, fraction [*a* + *b*], was significant at *α* = 0.05.

Our results suggest that while the geographical location does not appear to have a statistically significant impact on the adipogenic activity of crude extracts of localized* R. groenlandicum* samples, variations in active compounds do explain a significant proportion of variability in pharmacological activity. We have shown that annual temperature ranges and insolation parameters, such as solar radiation, could significantly explain some of the variation in the species' phenolic compounds [1]. Although we did not find in this study that these environmental variables could significantly explain the variation in TG content (*p* = 0.150, *R*_adj_^2^ = 0.162), we found, nonetheless, that they explained an important proportion of the variation in the phytochemical profiles of* R. groenlandicum*, which could significantly explain TG content (nontestable; see Table 1, Figure 6). Indeed, variation partitioning of TG content with both phytochemical and environmental variables indicates that while the relationship with compounds was statistically significant (*p* = 0.0279, *R*_adj_^2^ = 0.491), their unique contribution to explaining TG content no longer was when the contribution of environmental variables, albeit small, was taken into account and removed (*p* = 0.0619, *R*_adj_^2^ = 0.424).

This confirms the caveat that environmental variables play an underlying role in affecting the content of biologically active compounds. Conversely, quercetin, the only significant compound related to changes in the adipogenic activity of* R. groenlandicum*, was not found to be strongly associated with environmental variables [1]. This may hence explain why the portion of variation those environmental variables contribute to the model, and more specifically to the content of biologically active compounds, which best explains the variation in TG content, is considerably small (Table 1, Figure 6).

On the other hand, our results provide support for the hypothesis that synergistic interactions may occur between compounds. For instance, the inhibitory action of* Hibiscus sabdariffa* (Malvaceae) was greater than the sum of its parts when polyphenols had been fractionated, isolated, and tested individually [35]. More importantly, in bioassay-guided fractionation experiments, the activity of crude Labrador tea extracts was higher than that of each active compound tested individually [24]. Finally, quercetin and resveratrol, together, decreased lipid accumulation considerably more than each of these used separately at the same dose [34].

Many of the compounds quantified in this paper have shown adipogenic activity in some form or another. Quercetin-3-glucoside has been found toxic to adipocytes at relatively low concentration (50 *μ*M) but was not found to affect adipogenic activity [33]. Quercetin-3-rhamnoside has been found inactive at low concentrations yet inhibited adipogenesis at high concentrations and chlorogenic acid has also been found to inhibit intracellular triglycerides accumulation [33]. Content variations of some of these, particularly (+)-catechin and (−)-epicatechin, have been explained by environmental variables [1]. Although the individual effect of these compounds was not detected in our study, it does not undermine the role they may play when found in a cocktail of substances.

## 4. Conclusion

We have previously shown that latitude acted as a marker for the impact of environmental variables on phytochemical concentrations [1]. Therefore, a trend could possibly exist between abiotic factors and concentration of targeted secondary metabolites, but a larger sample size might be needed to detect it. There may also be other environmental, climatic, and even biotic factors that were not taken into account, which explain the changes in quercetin content. These may better explain the ecophysiological processes affecting the antidiabetic potential of* R. groenlandicum*. Increase in the adipogenic potential of this traditional medicine was associated primarily with lower concentrations of quercetin, but the cause for its variation will require further investigation. Nonetheless, our results do not provide enough evidence to justify the idea that specific accessions of Labrador tea may have reproducibly better adipogenic potential than others along a latitudinal gradient. Conversely, our study implies that random harvesting of* R. groenlandicum* in the Eeyouch territory of Northern Quebec should not have a major impact on the quality of traditional preparations or NHPs made from this plant.

## Figures and Tables

**Table 1:** Variation partitioning of the adipogenic activity of *R. groenlandicum *leaf extracts explained by the content in biologically active compounds (compounds) and the effect of bioclimatic variables (environment). Fraction [*a*] corresponds to the unique contribution of compounds once the environment has been taken into account, whereas fraction [*c*] represents the reverse. Fraction [*b*] represents the shared portion, or overlap, between the effect of compounds and environment. The variation (*R*_adj_^2^) of each fraction is represented in Figure 6. Asterisk (*∗*) indicates significant fractions at *α* = 0.05.

Fractions	*R*_adj_^2^	*p *
[*a* + *b*] = compounds	0.491	0.0279^*∗*^
[*b* + *c*] = environment	0.109	0.150
[*a* + *b* + *c*] = compounds + environment (full model)	0.534	0.0437^*∗*^
[*a*] = compounds∣environment	0.424	0.0619
[*b*] = shared	0.0668	Not testable
[*c*] = environment∣compounds	0.0424	0.302
[*d*] = unexplained (residuals)	0.466	Not testable
